# Effective therapeutic dosage of antipsychotic medications in patients with psychotic symptoms: Is there a racial difference?

**DOI:** 10.1186/1756-0500-1-25

**Published:** 2008-06-12

**Authors:** Muideen O Bakare

**Affiliations:** 1 Child and Adolescent Unit, Federal Neuro-Psychiatric Hospital, New Haven, Enugu, Enugu State, Nigeria

## Abstract

**Background:**

Genetic make up had been known to influence pharmacokinetics and pharmacodynamics of psychotropic medications. Time separation in evolutionary trend in Africans, Orientals and Caucasians had been thought a possible explanation for the observed racial variation in activities of Cytochrome P 450 (CYP 450) enzymes, which are responsible for metabolism of psychotropic and other medications in human. Past studies on pharmacokinetics and pharmacodynamics of antipsychotic medications and socio-cultural factors influencing response to antipsychotic medications had consistently giving an inkling of possible racial difference in symptoms response to antipsychotic medications. Another growing body of evidence subscribing to possible racial difference in psychotic symptoms response to antipsychotic medications is the observed variation in antipsychotic medications prescription pattern and dosage across races and regions. Empirical observation had shown that dosage prescription pattern of antipsychotic medications in most Sub-Saharan African countries deviates from the standard prescription guidelines published for use in western parts of the world. Studies coming from the United States (U.S) had consistently reported a higher dosage of antipsychotic medications prescription for African-American patients compared to their Caucasian counterparts. Research on East Asia Psychotropic Prescription (REAP) study had also identified high dosage antipsychotic medications prescription pattern well above the recommended dose of 1,000 mg Chlorpromazine equivalent per day as common practices in some East Asian countries.

**Presentation of the Hypothesis:**

The pertinent question is why the apparent differences in dosage prescription practices across races and regions? The possibility of racial differences in psychotic symptoms response to antipsychotic medications rather than clinicians' prescription attitudes was entertained.

**Testing the Hypothesis:**

Future carefully controlled studies might be needed to test the proposed hypothesis of racial differences in psychotic symptoms response to antipsychotic medications.

**Implication of the Hypothesis:**

There might be actual racial influence on psychotic symptoms response to antipsychotic medications. If future carefully controlled studies uphold the hypothesis of racial differences in psychotic symptoms response to antipsychotic medications, there might be need to draw up new treatment or prescription guidelines that would put into consideration variations in genetic make up and consequent racial differences in psychotic symptoms response to antipsychotic medications.

## Background

Genetic make-up had been known to influence metabolism and utilization of psychotropic medications [1-4]. Genetic and environmental factors also determined the phenotypic inter-racial differences worldwide. Earlier studies had documented racial variation in metabolism of antipsychotic medications based on serum level measurement of the medications and other parameters [5,6]. The findings of these studies [5,6] could be due to racial differences in activities of cytochrome P 450 (CYP 450) enzymes, especially CYP2D6 and CYP2C19 which are largely responsible for metabolism of antipsychotic medications and other psychotropic drugs [1-4]. Another possible factor that could be responsible for racial variation in serum level of antipsychotic medications and other parameters of psychotic symptoms response is individual variation in dopamine receptor occupancy of antipsychotic medications which had been documented [7].

In the past two decades, review of literature on pharmacokinetics and pharmacodynamics of psychotropic medications and socio-cultural influences on response to psychotropic medications in different races had consistently pointed an exploratory search light at possible variation in racial symptoms response to psychotropic medications [8-10]. Various determinants ranging from biological to socio-cultural factors had been proposed as explanations for the often observed racial difference in metabolism and response to psychotropic medications [10]. Aside the biological and socio-cultural explanations put forward by these reviews as regards psychotropic medications in general [10], variation across races and ethnicity in the prescription pattern and dosage of antipsychotic medications is another growing body of evidence subscribing to the possible existence of variation in racial or ethnic symptoms response to antipsychotic medications in particular.

Prescription pattern and dosages of antipsychotic medications prescribed varies from one patient to another, from one professional to another, from one clinical institution to another and from one region to another [11]. Empirical observation and few studies available in Nigeria [12,13] had shown that antipsychotic prescription pattern and dosages of antipsychotic medications prescribed in Sub-Saharan Africa deviate from the prescription guidelines published for use in western parts of the world [14,15]. High dosage of antipsychotic medications are often prescribed in Sub-Saharan Africa and use of depot antipsychotic medications are common [13], when compared to recommendations in prescription guidelines published for standard psychiatric practice in western countries of the world [14,15].

It had also been found that African-Americans compared to their Caucasian counterparts often received antipsychotic medications for a longer period of time as maintenance treatment for Bipolar Affective Disorders (BAD) [16,17]. Both studies [16,17] could not adduce any particular reason for this difference between African-American patients and their Caucasian counterparts. Walkup et al [18] also found that African-Americans were more likely to be prescribed higher dose of antipsychotic medications than the recommended dosage (a dose greater than 1,000 mg Chlorpromazine equivalent per day), they concluded that there was a tendency for minority individuals to be prescribed higher dosage of antipsychotic medications in excess of the recommended range. Diaz and De Leon [19] had found that the odd of being prescribed excessive doses of typical and high potency antipsychotic medications is about two times higher for African-American than Caucasian patients with schizophrenia.

## Presentation of the hypothesis

### Hypothesis of racial differences in effective therapeutic dosage of antipsychotic medications in treating psychotic symptoms

The reasons for the difference between the prescription pattern in African-American and their Caucasian counterparts in above studies [16-18] could not be adequately explained and the observation that minority individuals were likely to be prescribed higher doses of antipsychotic medications did not give a satisfactory answer to why the difference?

Therefore, a possible explanation is that at the same level of severity of psychotic symptoms as measured by any standardized rating scale, African-Americans and black Africans of Sub-Saharan African descent might require a higher dosage of antipsychotic medications compared to their Caucasian counterparts for resolution of their psychotic symptoms. This difference may lie in varied pharmacogenetic effects in metabolism and utilization of antipsychotic medications in different races of the world.

The argument of racial differences as it affects the effective therapeutic dosage of antipsychotic medications in treatment of psychosis is given a further weight by Sim et al study [20], which reported higher dosage of antipsychotic medications prescription pattern in East Asian countries, especially Japan, Korea and Singapore.

There is need to explore the possible explanations for the apparent racial and regional differences in dosage prescription pattern of antipsychotic medications.

### Differences in prescribing practices

It had already been noted that prescribing practices might vary from one professional to another and from one region to another [11]. This may be because of variation in training background of individual mental health professionals. It may also be differing practices in different regions of the world which had come to stay because of empirical observation in practice over a long period of time that symptoms resolution of psychosis are delayed or unachievable when the recommended guide lines are strictly adhered to. This may be the possible picture of situation in Sub-Saharan African countries where empirical observation had shown higher dose of antipsychotic medications prescription among mental health professionals compared to the western world.

The possible argument against differences in prescribing practices being a function of training background could be found in literature coming from the United States (US), where training of mental health professionals is regulated by a uniform body and can be presumed to be a uniform training background. In these literatures [16-18], it had been consistently found that African-American are likely to be placed on higher dose of antipsychotic medications and are likely to take same as maintenance medications over a longer period of time compared to their Caucasian counterparts. It could also be argued that the relative higher dosage of antipsychotic medications being prescribed in African-Americans compared to their Caucasian counterparts is borne out of a long standing practicing norms derived from empirical observations, which had not been subjected to evidence based practice approach through carefully controlled studies.

Therefore, the significant difference in dosage prescription pattern and longer term use of antipsychotic medications observed in African-Americans and black Africans in Sub-Saharan Africa compared to their Caucasian counterparts may not be solely explained by variation in training backgrounds of attending mental health professionals in these regions of the world.

### Socio-cultural acceptability and unacceptability of the presenting psychotic symptoms

Socio-cultural acceptability of patients with symptoms of mental illness may vary according to symptoms presentation. Patients with depressive symptoms are likely to be more accepted and coped with than patients that present with psychotic symptoms, while patients that present with psychotic symptoms without aggression are tend to be more accepted and coped with than patients that present with psychotic symptoms and aggression.

The socio-cultural acceptability and unacceptability of patients' symptoms affect directly and indirectly the prescription pattern and practices. Famuyiwa [12] noted that there was tendency for patients with depression to be under treated or under medicated, possibly because of high acceptability of these patients compared to those presenting with psychotic symptoms. Sim et al [20] had identified aggression as an important factor positively associated with high dosage prescription of antipsychotic medications in East Asian region. The relevance of socio-cultural acceptability and unacceptability of symptoms presentation as it affects prescription practices is further highlighted from the report of Barbui et al study [21], who found that positive symptoms presentation in patients with schizophrenia was positively associated with high antipsychotic dose prescription, whereas negative symptoms presentation was negatively associated with high antipsychotic dose prescription above recommended prescription guidelines.

Relating Barbui et al [21] findings to the findings of Diaz and De Leon [19] who found that the probability of being prescribed high dose and high potency antipsychotic medications was about two times higher for African-Americans than for Caucasian patients with schizophrenia, one would be tempted to ask whether African-Americans are likely to present with more florid positive symptoms of schizophrenia compared to their Caucasian counterparts. Another plausible explanation is that African-American patients presenting with psychotic symptoms were usually assessed to be more potentially aggressive and dangerous compared to their Caucasian counterparts. These two possibilities may be able to explain the apparent difference in antipsychotic medication dosage prescription pattern for African-Americans and their Caucasian counterparts.

Then, the zeal to curtail aggression and potential dangerousness in a patient presenting with psychotic symptoms may be the reason behind the dosage prescription of antipsychotic medication well above the recommendation in standard prescription guidelines. This may also explain poly-pharmacy and common use of depot antipsychotic medications among African-American and black African patients from sub-Saharan Africa that had been noted in some studies [13,18-21].

### Hypothesis of racial differences in psychotic symptoms response to antipsychotic medications

Another possible explanation for differences in dosage prescription pattern of antipsychotic medications that had been found in the above studies [11,13,16-21] is racial differences in psychotic symptoms response to various antipsychotic medications.

Having been established that genetic variation have some influence on pharmacodynamics and pharmacokinetics of psychotropic medications [1-4], there are possibilities that racial differences in genetic make-up may influence the effective therapeutic response to dosage of antipsychotic medications.

The deviation in dosage prescription practices from prescription guidelines published for use in western parts of the world [14,15] in most Sub-Saharan African countries and the consistent findings of studies coming from the United States [11,16-19,21,22], that documented high dosage prescription of antipsychotic medications among African-American patients compared to their Caucasian counterparts and also the study by Sim et al [20] coming from East Asia give some support to the hypothesis that racial differences in psychotic symptoms response to antipsychotic medications might be responsible for the apparent difference in dosage prescription practices. Although, Diaz and De Leon [19] were quick to conclude that pharmacogenetic differences were unlikely to explain the significant racial difference in dosage prescription pattern of antipsychotic medications found in their study and adduced reason for their finding to clinicians' attitude. However, the argument that clinicians' attitude could be responsible for their finding need to be subjected to further challenge in the presence of growing body of evidence supporting biological and socio-cultural determinants for possible ethnic or racial difference in response to psychotropic medications [1-4,8-10]. This would explain why the authors themselves [19] recommended pharmacogenetic testing in future studies to unravel the reason for their finding which had been consistently replicated by other studies [11,16-18,21,22].

## Testing the Hypothesis

Considering the presence of other confounding factors like availability and cost of antipsychotic medications, health care financing systems among other factors which may vary in different regions of the world and can contribute to the observed difference in prescription pattern and dosage of antipsychotic medications across races and regions, the need for further studies is apparent to confirm or refute the hypothesis of racial difference in psychotic symptoms response to antipsychotic medications. These studies would be necessary to unravel the actual reason behind the observed difference in dosage of antipsychotic medication prescription that existed among black Africans, African-Americans, Orientals and their Caucasian counterparts. Controlled studies, utilizing pharmacogenetic testing, standardized psychotic symptoms resolution assessment method and serum level measurements of antipsychotic medications during treatment might be able to provide a definitive answer to the puzzling question of observed differences in dosage of antipsychotic medication prescription across races and regions. Actual racial differences in psychotic symptoms response to antipsychotic medications due to genetic variations might be the underpinning factor.

## Implication of the Hypothesis

If the hypothesis of racial differences in psychotic symptoms response to antipsychotic medications is upheld by future studies, there might be need to draw up new prescription or treatment guidelines that would take into consideration variations in genetic make-up and consequent racial differences in psychotic symptoms response to antipsychotic medications.

## Competing interests

The author declares that they have no competing interests.

## Authors' contributions

MOB conceived and wrote the manuscript. The author read and approved the final draft of the manuscript.
